# Effect of restaurant consumers’ anticipated emotions on perceived value and behavioral intention in the COVID-19 context

**DOI:** 10.3389/fpsyg.2022.1013209

**Published:** 2022-12-22

**Authors:** Yanmei Jiang, Antonio K. W. Lau

**Affiliations:** ^1^The School of Business, Anhui University of Technology, Ma’anshan, China; ^2^Key Laboratory of Multidisciplinary Management and Control of Complex Systems of Anhui Higher Education Institutes, Anhui University of Technology, Ma’anshan, China; ^3^The School of Management, Kyung Hee University, Seoul, South Korea

**Keywords:** perceived value, perceived threat, dining out intention, COVID-19, anticipated emotion

## Abstract

While hospitality scholars have been conducting research on post-pandemic consumption recovery, the impact of the psychological consequences of COVID-19 on consumers’ post-pandemic behavior remains insufficiently addressed. Therefore, the purpose of this study is to explore the relationships among anticipated emotions, perceived value, perceived threat, and dining-out intention in the COVID-19 context. In this study, 621 restaurant consumers in China were surveyed and the data were analyzed with structural equation modeling. The results suggested that positive anticipated emotions affect perceived hedonic, utilitarian, and social values, whereas negative anticipated emotions affect hedonic value. Hedonic and utilitarian values then influence dining-out intention. Perceived threat in terms of perceived severity and susceptibility to COVID-19 was explored to moderate the impacts of hedonic and social values on the intention. This study contributes to the literature by identifying the positive, distinct roles of both anticipated emotions on consumer dining-out intention through perceived values and threats during the early recovery of COVID-19.

## Introduction

The tourism and hospitality industries are among the hardest-hit sectors in the global economy due to the COVID-19 pandemic (UNWTO, 2021, 2022). With the spread of vaccines and the need for economic development, governments and organizations across the world are actively promoting the recovery of tourism and hospitality, such as easing restrictions and learning to live with COVID-19 (Geducos, 2022). Meanwhile, the academic community has been conducting research on this post-pandemic recovery, especially for the improvement of hygiene and the encouragement of consumption in the hospitality industry. Zhong et al. (2021), for example, explored the key drivers, including restaurant precautionary hygiene measures, of individuals’ dining-out intentions. Liu et al. (2021a) investigated the factors influencing post-pandemic outbound travel, such as non-pharmaceutical interventions. However, the impact of the psychological consequences of COVID-19 on consumers’ post-pandemic behavior remains insufficiently addressed in the literature (Hassan and Soliman, 2021; Blazquez-Resino et al., 2022).

To prevent the spread of COVID-19 initially, governments adopted strict prevention policies, including social distancing, self-quarantine, closing shops, banning dining in restaurants, limiting private gatherings, and directly blocking certain severely affected areas (BBC, 2021). In such a social environment, customer demand was suppressed, which might have changed their attitudes and psychological behaviors (Baculinao et al., 2020; Seyitoğlu and Ivanov, 2021). After easing of restrictions, consumers should be eager to return to normal life, such as dining in a restaurant as usual before the outbreak. However, as the pandemic has not been completely under control and the virus is constantly mutating (WHO, 2022a), thousands of people are still infected every day (WHO, 2022b). The annual GDP growth of the restaurant industry in G20 countries reduced by approximately 2% in 2020 (Mac Donald et al., 2020). With the economic downturn, people are now less likely to dine out now as they have become used to online delivery (Mahmood et al., 2022) or cooking more at home (Ko et al., 2022). Therefore, it is crucial for hospitability scholars to understand the consumer psychological states in the recovery stage of COVID-19 and how such psychological effects influence consumption behaviors, which remains underexplored in the literature (Mahmood et al., 2022).

Moreover, the mediation mechanisms between emotion and behavioral intention under the new normal context of COVID-19 (Chiu and Cho, 2022) have not been well-examined in restaurant studies (Han et al., 2019b). Some scholars have suggested that perceived value is a key mediator between emotion and behavioral intention, as it is regarded as a comparison result of perceived benefit and sacrifice (Zeithaml, 1988), which can also result from the changes in consumers’ emotional behavior (Liu and Jang, 2009). Perceived value can be hedonic and utilitarian values (Ha and Jang, 2010; Byun and Mann, 2011; Song and Qu, 2017; Choi et al., 2020a) or social value (Kim et al., 2019). However, the existing literature has rarely integrated these three types of perceived values in a single study and inspected their distinctive roles in the relationships between consumer emotions and dining-out intention in the context of COVID-19.

Furthermore, recent research has called for more studies on how consumers’ perceived threat of the pandemic impacts behavioral intentions (Kirk and Rifkin, 2020; Laato et al., 2020). An individual’s perceived threat, measured by perceived severity and susceptibility, can affect their consumption patterns and behavioral intention (Liu et al., 2021b). For instance, if the perceived threat of the COVID-19 pandemic increases, an individual’s variety-seeking intention will be strengthened across different brands but weakened within the same brand (Kim, 2020). Campbell and Goodstein (2001) showed that the perceived threat is an important situational factor in an individual’s evaluation in the consumption context. High perceived threat leads consumers to become more conservative, while low perceived threat makes them evaluate products more positively. However, it remains unclear whether consumers’ perceived threat of COVID-19 can moderate the effect of their psychological state on behavioral intention in the new COVID-19 context (Peng and Chen, 2021). Therefore, the research questions of this study are as follows:

1. How does the anticipated emotions of restaurant consumers influence their behavioral intention through perceived value in the context of COVID-19 recovery?
2. How does the perceived threat of COVID-19 affect the relationship between perceived value and behavioral intention in this context?

This study aims to explore the relationships among anticipated emotions, perceived value, perceived threat, and dining-out intention in the context of the COVID-19 pandemic. In particular, the study has the following objectives: (1) investigate the distinctive effects of positive and negative anticipated emotions on dining-out intention through three perceived hedonic, utilitarian, and social values; (2) examine the relative importance of the three perceived values on behavioral intention; and (3) explore the moderating effects of perceived threat on these relationships.

This study contributes to the extant literature in four ways. First, the role of consumer emotions affected by a social stimulus or a forced behavior (e.g., social distancing due to the outbreak of COVID-19), rather than a marketing stimulus typically studied in the consumer behavior literature on behavioral intention, is explored (Babin and Attaway, 2000; Ivascu et al., 2022). This study is expected to expand the existing literature on how a socially stimulated emotion affects consumer behaviors (Hassan and Soliman, 2021; Zhong et al., 2021). Furthermore, the distinctive effects of positive and negative anticipated emotions on dining-out intention through perceived value are examined. It contributes to the consumer psychology literature by providing new empirical evidence to support the unique roles of both anticipated emotions on consumer behaviors (Bagozzi et al., 1998; Taylor et al., 2016; Foroudi et al., 2021). Specifically, this study verifies that both positive emotions to dine out and negative emotions to not dine out can drive consumer behaviors through the three perceived values. Second, the internal mechanisms between anticipated emotions and behavioral intention are examined through the mediation effect of the three perceived values—hedonic, utilitarian, and social—simultaneously (Peng and Chen, 2021). Thus, new evidence is provided to examine the distinguishing roles of these perceived values in the relationship of emotions and behavioral intention under a new pandemic context. Third, this is one of the first empirical studies to verify the moderating effect of perceived threat on the relationship between perceived value and behavioral intention (Cahyanto et al., 2016), which is insufficiently studied in the COVID-19 context^1^ (Laato et al., 2020; Kim et al., 2021; Li et al., 2022). Thus, this study can provide valuable insights into consumer behavioral intentions from the health perception perspective. Finally, this study provides new ideas for the tourism and hospitality industries to recover from COVID-19. This paper proposes that the anticipated emotions to dine out after the COVID-19 recovery can affect how consumers perceive the hedonic, social, and utilitarian values of going to restaurants, leading to dine-out intention. The results of this study can help advice practitioners on resource prioritization for enhancing different perceived values and anticipated emotions to encourage dine-in behaviors. For example, if only positive emotions were found to affect perceived value, the manager may promote a pleasured and familiar dining environment to the consumers’ thought engagement and social media (Haas et al., 2022) while not advertising the missed opportunities of not dining out. By assessing the impact of perceived COVID-19 threat, the regulators or other stakeholders on whether and how the perceived threat plays a role in it are also recommended (Kim et al., 2021; Zhong et al., 2021), which is important for the recovery of hospitality and restaurant industries (Mahmood et al., 2022; Sardar et al., 2022).

The remainder of the paper is organized as follows. First, a relevant literature review is provided. Then, the research model along with five hypotheses is presented. The methodology and main results are discussed later. Finally, the findings are concluded with theoretical contributions and managerial implications.

## Literature review

### Anticipated emotions in purchasing decisions

Anticipated emotions can be defined as “*predictions of an outcome’s emotional consequences or beliefs about one’s own emotional responses to future outcomes*” (Bagozzi et al., 2016, p. 630). They play a crucial role in predicting individual behavior (Mellers and McGraw, 2001; Conner et al., 2013; Bagozzi et al., 2016; Taylor et al., 2016; Ahmed and Ting, 2020). The key process of anticipated emotions is about forward-looking counterfactual thinking processes (Mandel et al., 2005). In this process, a consumer imagines the possible results and what emotional reactions would be experienced before they decide or take action (Feil et al., 2022). The alternative consequences of the success or failure of an expected action serve as inputs to the evaluation process and emotional experience (Bagozzi et al., 1999). An individual is encouraged to choose actions that promote positive emotional experiences or avoid negative ones related to their needs being met or not (Mellers and McGraw, 2001). The actions chosen are mainly affected by the relative efficacy and coexistence of positive and negative emotions (Manthiou et al., 2020). Exemplars are anticipated pleasure, guilt, frustration, pride, and regret (Kim et al., 2013a; Kotabe et al., 2019; Haj-Salem et al., 2022). Thus, anticipated emotions broadly involve two dimensions: positive anticipated emotions and negative anticipated emotions (Song and Qu, 2017; Ahmed and Ting, 2020), which are distinguishable and useful for understanding consumer reaction or behavioral intention (Babin and Attaway, 2000; Bagozzi et al., 2016). Early studies on anticipated emotions have shown that negative affective reactions in the forms of anticipated regret have a stronger effect than positive anticipated emotions on behavioral change (Abraham and Sheeran, 2004; Conner et al., 2013; Bettiga and Lamberti, 2020). To affect purchasing decisions, both positive and negative anticipated emotions can be separated by motivating purchase and motivating non-purchase [Bagozzi et al., 2016; i.e., positive anticipated emotions to purchase (situation 1), negative anticipated emotions to purchase (situation 2), positive anticipated emotions to non-purchase (situation 3), and negative anticipated emotions to non-purchase (situation 4)]. When situations 1 and 4 occur co-instantly in a positive way, they motivate purchase, and when situations 2 and 3 co-occur positively, they motivate non-purchase. As this study aims to explore the role of emotions in motivating restaurant visits, the positive emotions to dine out and negative emotions not to dine-out situations are examined, and this examination is consistent with existing hospitality and tourism literature (e.g., Shin et al., 2018; Ahn and Kwon, 2020).

Recently, the anticipated emotions have been frequently discussed with the model of goal-directed behavior (MGB), which is an extension of the theory of planned behavior (TPB). TPB suggests that human behaviors are caused by intention and actual behavioral control, resulting from attitude toward the behavior, subjective norm, and perceived behavioral control, which represent behavioral, normative, and control beliefs, respectively (Ajzen and Kruglanski, 2019). This theory is highly popular in hospitality and tourism research (Ulker-Demirel and Ciftci, 2020). MGB extends TPB by adding positive and negative anticipated emotions as key predictors in the TPB model with desire on purchase intention (Chiu and Cho, 2022). It argues that both positive and negative anticipated emotions can help explain consumer behaviors with TPB constructs (Bagozzi et al., 1998, 2016); in contrast, in a meta-analysis of 37 empirical studies, the anticipated emotions are found to strongly affect the desire for behavioral intention and actual behavior (Chiu and Cho, 2022). Controlling for the TPB constructs, negative anticipated emotions reduce consumers’ expectation of behaving negatively (Moan and Rise, 2005; Odou and Schill, 2020). The anticipated emotions can affect the intention to purchase innovative products (Seegebarth et al., 2019), purchase intention toward electric vehicles (He et al., 2022), and pro-environmental behaviors (Han et al., 2019a; Odou and Schill, 2020; Zhao et al., 2020). In the COVID-19 situation, the anticipated emotions can improve the consumer’s sense of obligation to exhibit pro-social behaviors, such as wearing masks, keeping social distance, and practicing sanitation activities (Chi et al., 2021). Positive and negative anticipated emotions are important factors for early traveling decisions after the pandemic (Wang et al., 2021). They are critical and distinguishable and can predict behavior evaluations (Richetin et al., 2008; Taylor et al., 2016; Londono et al., 2017; Chiu and Cho, 2022). Recent studies have shown that, compared to TPB constructs, anticipated emotions play major roles in behavior intention, for example, for breastfeeding (Parkinson et al., 2018), energy-saving (Wang et al., 2018), and visiting oriental medicine festivals (Song et al., 2014).

In addition, some scholars have suggested that the role of emotions in behavioral intention can be studied with the stimulus (S)-organism (O)-response (R) paradigm, which explains the effect of emotions (O) generated from the stimulus (S) on behavioral intention (R; Mehrabian and Russell, 1974; Liu and Jang, 2009). Internal and external stimuli can create emotions that affect a customer’s perception of service value, which is a cognitive judgment of perceived benefit and cost (Bitner, 1992). Positive emotions generate emotional benefits for consumers to positively evaluate services and stay longer to enjoy them, while negative emotions create an emotional cost for them to lower their purchase involvement and withdraw from the services (Liu and Jang, 2009; Byun and Mann, 2011). Some psychology scholars have also suggested that emotions are a type of affective information that positive emotions leads one to go out and explore, while negative emotions lead one to stay vigilant and cautious (Zadra and Clore, 2011). For example, people with positive anticipated emotions tend to take riskier behaviors, such as binge-drinking (Carrera et al., 2012). Following the S-O-R paradigm and consistent with the TPB/MGB concepts, this study proposes that the early recovery of COVID-19 where restaurants were reopened is an external stimulus, and this stimulus can affect the anticipated emotions which change the cognitive evaluation of the perceived values to dine out, leading to dining-out intention.

#### Anticipated emotions in a restaurant setting

In the restaurant literature, anticipated emotions have been studied with different measures. For example, anticipated emotions in terms of the anticipated regret can affect the intention to select an eco-friendly restaurant (Kim et al., 2013b). Anticipated pleasure, but not anticipated guilt, directly influences healthy food consumption in a quick service restaurant setting (Hur and Jang, 2015). Anticipated pride with waste reduction and anticipated guilt without waste reduction can affect the diner’s intention to reduce waste at a restaurant (Kim and Hall, 2019). Consumers with anticipated pleasure generated from healthy advertising appeals may prefer plant-based menu items at restaurants (Ye and Mattila, 2021). Positive anticipated emotions generated from the perceived brand relationship orientation can improve hotel brand performance (Casidy et al., 2018). The physical environment induces positive anticipated emotions, which improve the consumer’s willingness to participate in the co-creation experience in a restaurant (Im et al., 2021). Positive and negative anticipated emotions affect consumers’ revisit intention to green hotels (Ahn and Kwon, 2020; Kwon and Ahn, 2020). Foroudi et al. (2021) showed that, with the COVID-19 shock, the positive emotions can increase the consumer’s desire for future restaurant visits even if a lockdown policy is implemented (see also Núñez-Fernández et al., 2021). According to the literature search of this study, recent studies have mainly adapted the broad views of emotions (i.e., positive and negative emotions) without addressing different discrete emotions (Perugini and Bagozzi, 2001; Lee et al., 2012), which this study also follows.

In the face of COVID-19, consumers may have different responses to dining behaviors (Min et al., 2021). For example, in India, diners might look for food quality, price, and hygienic practices in a restaurant (Lim et al., 2022). In Korea, their subjective norm and behavioral control to dine out encourage them to visit restaurants, but the psychological risk discourages them (Zhong et al., 2021). In China, although the local pandemic was brought under control at the time of this study, the numbers of confirmed cases and deaths around the world quickly rose every day during the same period (WHO, 2021). However, long-term home isolation and lockdown might make people more eager to go out and interact with others (BBC, 2020a), such as enjoying a tasty meal and having a good time with friends in a restaurant. Thus, it is interesting to understand consumers’ mental state and how psychological emotions affect their evaluation and intention of dining behaviors in this new context.

### Perceived value

Perceived value refers to “*the consumers’ overall assessment of the utility of a product based on perceptions of what is received and what is given*” (Zeithaml, 1988, p. 14). It is multidimensional, situational, and context-dependent, covering both cognitive and intrinsic aspects of decision-making (Sweeney and Soutar, 2001; Sánchez-Fernández and Iniesta-Bonillo, 2007), comprising different types of perceived values (Kim et al., 2019). Recent literature has suggested that hedonic and utilitarian values are two critical elements of perceived value in consumer behaviors (Yu et al., 2013; Song and Qu, 2017). Hedonic value refers to the amusement and emotional worth of the consumption experience, whereas utilitarian value represents the usefulness of consumption in efficient, task-related, functional, and economic aspects (Babin et al., 1994). Hyun et al. (2011) found that advertising-induced emotional responses positively affect patrons’ hedonic and utilitarian values in the chain restaurant industry. Hedonic and utilitarian values can provoke impulse buying of daily deals (Yi and Jai, 2020) and motivate the use of food trucks (Shin et al., 2018) and sustainable restaurant practices (Kim and Hall, 2020). The values stimulate consumers to use onsite restaurant-interactive self-service technology (Xu et al., 2020) and can be triggered by promotional cues (Hlee et al., 2019) and the restaurant retailing structure (Brown, 2020).

Social value is another important element of perceived value because consumers may evaluate a product based on the social outcomes of what it communicates to other people (Sweeney and Soutar, 2001). Social value is the perception of value generated from advancing, expanding, and preserving relationships through self-expression, communication, and interactions with other people (Zeithaml, 1988). It enhances a person’s self-concept and gratification in one’s social relationship (Yu et al., 2013) through social status and self-esteem (Rintamäki et al., 2006). Thus, social value is consequential and independent of hedonic and utilitarian values, as the former is related to group referents, while the latter two refer to individual affective responses for their own purpose (Akram et al., 2021). Some e-business scholars have confirmed the significance of social value in online and offline purchases (e.g., Wu et al., 2018; Yu and Huang, 2022). During the COVID-19 situation, social benefits from interpersonal communications could improve the social connection between consumers and other people, generating a better customer experience and intention to purchase (Zhang and Zhang, 2022).

In the case of this study, as consumers’ normal social activities might not be conducted due to the social distancing restrictions of the pandemic, they may be more eager for normal consumption life and social activities in the early recovery of COVID-19. Dining in a restaurant involves considerable social value for consumers (Park, 2004), which cannot be ignored, especially in the context of COVID-19, which involves social distancing practices (Lim et al., 2022; Mahmood et al., 2022). However, recent literature has not studied hedonic, utilitarian, and social values simultaneously (Kakar, 2020) in the context of COVID-19 (Zhang and Zhang, 2022), which warrants further studies.

## Research model and hypothesis

**Figure 1:**
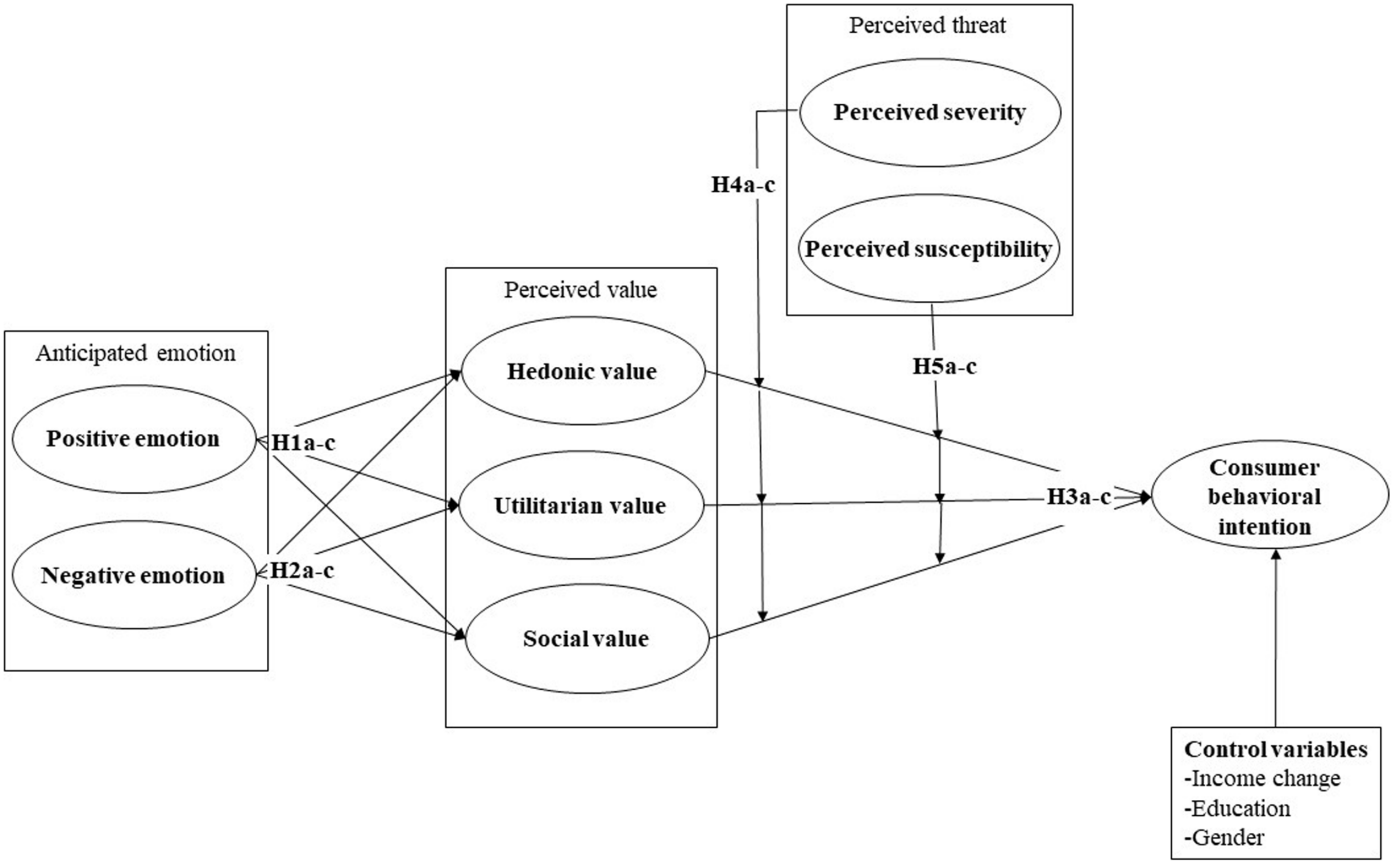
Research model.

Based on the literature review, this paper proposes a research model, as shown in Figure 1. It hypothesizes that a restaurant consumer’s positive and negative anticipated emotions influence perceived values (i.e., hedonic, utilitarian, and social values), which in turn affects the intention of dining in a restaurant. In addition, consumers’ perceived threat of the pandemic (i.e., perceived severity and susceptibility) can moderate the effects of perceived value on the dine-in intention.

### Roles of emotions, perceived value, and behavioral intention

A person’s emotional state can affect their information processing processes, including the encoding and retrieval of information, forming strategies for processing information, evaluation and judgment, and creative thinking (Bagozzi et al., 1999). Previous studies have shown that individuals in positive emotional states rate the stimuli (e.g., people, consumer goods, or service and life events) more positively than people in neutral or negative emotional states (Hyun et al., 2011). Individuals in a positive affective state are motivated to maintain their good mood or emotions (Wegener et al., 1995; Bagozzi et al., 1999), avoid systematic processing of information, and resort to less demanding heuristic processing. Thus, positive anticipated emotions play a positive role in the information processing and evaluation of a product, which should increase the perceived value of the goods or services. This paper proposes that positive anticipated emotions, such as joy and happiness, will encourage consumers to make a more positive evaluation of the perceived value of dining out. The perceived values for dining in a restaurant include hedonic value, representing the good subjective experiential aspects, and utilitarian value, reflecting the economical and efficient aspects of dining out (Park, 2004; Hyun et al., 2011; Song and Qu, 2017). Some empirical studies have suggested that positive anticipated emotion has a positive effect on hedonic and utilitarian values (e.g., Hyun et al., 2011; Lo and Wu, 2014; Choi et al., 2020a). Positive emotions, including joy and happiness, increase hedonic shopping value, while negative emotions, including anger, reduce the value (Byun and Mann, 2011). Positive emotions, including joy, happiness, and delight, affect the subjective evaluation of the service that improves the utilitarian value of a flight trip (Choi et al., 2020a), or the hedonic value of an amusement park visit (Huseynov et al., 2020). Positive anticipated emotions, including happiness and positive affect, and negative anticipated emotions, including fear and guilt, can enhance hedonic and utilitarian attitudes for brand loyalty intention (Taylor et al., 2016). In addition, when dining out, restaurant consumers may pursue social value to satisfy their need for social relationships and mood transformation (Park, 2004). Thus, the anticipated emotions can also enhance the perception of the social value of dining in.

While the causal relationships between emotions and perceived value are still mixed (Song and Qu, 2017; Jeong et al., 2020), anticipated emotions can be one of the factors that affect the overall evaluation of one’s hedonic, social, and utilitarian experiences (Yüksel and Yüksel, 2007). Consumers may evaluate the perceived utilitarian (e.g., low price), hedonic (e.g., enjoyable dining experience), and social (e.g., meeting people in a restaurant) values more positively due to the positive affective information (Zadra and Clore, 2011) or emotional benefits (Liu and Jang, 2009) derived from positive anticipated emotions. For example, Kwak et al. (2011) reported that the anticipated emotions stimulated from promotional messages can improve the perceived value of a sport consumer’s purchase intention as it can shape the cognitive evaluation of a product. Thus, this paper proposes the first set of hypotheses:

*H1a*: Positive anticipated emotions are positively associated with hedonic value.

*H1b*: Positive anticipated emotions are positively associated with utilitarian value.

*H1c*: Positive anticipated emotions are positively associated with social value.

Individuals consider the emotional consequences of their actions or non-actions before they make any decision (Bagozzi et al., 2016). Seeking pleasure (i.e., positive emotions) and avoiding pain (i.e., negative emotions) are basic human motivations in one’s life (Higgins, 1997). When people make a purchase decision, they may avoid unfavorable non-purchase or delayed purchase feelings (Zeelenberg et al., 2000). Thus, people may process to positively evaluate a product or service to avoid negative outcomes (Bagozzi et al., 2016). In the context of dining out, to avoid the negative anticipated emotions of not being able to dine out, consumers may evaluate the perceived utilitarian (e.g., convenience, food, and service quality), hedonic (e.g., pleasant dining atmosphere), and social (e.g., social interactions provided by a restaurant) values at a higher level. In other words, the consumers may imagine bias on highly evaluating the convenience, food and service quality, pleasant dining atmosphere, and social interactions provided by restaurants. Alternatively, negative emotions may play a smaller role in purchasing decisions as consumers can simply avoid the evaluation (Zeelenberg et al., 2000). For instance, in the context of the COVID-19 pandemic, Foroudi et al. (2021) found that negative anticipated emotions have an insignificant effect on a diner’s future desire. Thus, consistent with H1, the following hypotheses are proposed:

*H2a*: Negative anticipated emotions are positively associated with hedonic value.

*H2b*: Negative anticipated emotions are positively associated with utilitarian value.

*H2c*: Negative anticipated emotions are positively associated with social value.

Perceived value is considered a critical predictor of consumers’ behavior intention in hospitality literature (Ha and Jang, 2010; Song and Qu, 2017). For example, in the casual restaurant industry, restaurant image significantly affects perceived value, which in turn guides restaurant consumers’ behavioral intention (Ryu et al., 2008), and perceived utilitarian value has a greater impact on behavioral intention than hedonic value (Ryu et al., 2010). In the chain restaurant industry, advertising-induced emotional responses positively affect consumers’ hedonic and utilitarian values, which lead to behavioral intention (Hyun et al., 2011). In the context of Korean grocerant, the perceived functional and hedonic values can affect consumers’ behavioral intention, but hedonic value is more influential (Kim et al., 2019). Other studies have shown the positive effects of hedonic and utilitarian values on behavioral intention (Konuk, 2019). However, only a few studies have explored the relative effects of hedonic, utilitarian, and social values on restaurant consumers’ behavioral intention under the new normal context of COVID-19. Strict preventive measures may alter a consumer’s view on the hedonic value of a restaurant’s comfortable atmosphere and the social value of interacting with friends (Zhong et al., 2021; Lim et al., 2022), changing their intention of dining in a restaurant. Thus, the following hypotheses are proposed:

*H3a*: Hedonic value positively affects behavioral intention.

*H3b*: Utilitarian value positively affects behavioral intention.

*H3c*: Social value positively affects behavioral intention.

### Roles of perceived threat

To assess the roles of perceived threat of COVID-19 on behavioral intention, this study adopts the concepts of perceived severity and susceptibility from the health belief model (HBM) (Glanz et al., 2008). The HBM is widely used to explain why and under what conditions individuals engage in risk-reducing health behaviors (Champion, 1984; Ng et al., 2009; Su et al., 2021). According to the HBM, individuals are likely to take actions (e.g., taking a vaccine) if they feel susceptible to an illness or a condition, believe that the disease or condition would have serious potential consequences, and perceive that the anticipated benefits of taking a preventive action outweigh its costs (Glanz et al., 2008). Perceived severity is an individual’s belief about the seriousness of a condition or disease (e.g., COVID symptoms) and its potential consequences (e.g., death or the post-COVID conditions), whereas perceived susceptibility is an individual’s belief about the opportunities of undergoing a risk or getting a condition or disease (e.g., getting COVID; Glanz et al., 2008). Both are partly dependent upon the individual’s knowledge of the illness and have a strong cognitive component; they can be grouped to be called perceived threat (Janz and Becker, 1984).

Existing research recognizes that the perceived threat of diseases, including COVID-19, encourages vaccination intention (Su et al., 2021) but negatively influences consumer tourism or dining-out intention (Pine and McKercher, 2004; Zhong et al., 2021; Apaolaza et al., 2022). In the new normal context of COVID-19, consumers might be eager to return to their normal life to satisfy their pent-up demands when governments eased preventive measures to promote economic recovery (Geducos, 2022). However, as the pandemic has not yet been fully contained, individuals are still at risk of infection (WHO, 2022b). In other words, perceived threat may cause consumers to reduce their behavioral intention or avoid dining out. For example, the high perceived severity and susceptibility of getting COVID-19 elevates self-protection behaviors by avoiding knowledge-sharing with colleagues (Wang et al., 2022), reducing traveling (Zheng et al., 2022), or adopting multiple social distancing practices, such as keeping away from public gatherings, doing most of individual activities at home, and using more online media for interpersonal communications (Itani and Hollebeek, 2021). In this way, consumers with a high level of perceived threat in COVID-19 require additional motivational factors (i.e., a higher level of perceived values) to push them to get out of home and meet people, like dining out (Huang and Wyer Jr, 2015; Kim, 2020; Itani and Hollebeek, 2021; Zhong et al., 2021). Thus, a higher perceived value of dining out is necessary to lure customers back to the restaurants. When restaurant consumers perceive a high level of perceived severity and susceptibility to the pandemic, it is more critical for them to realize the perceived value to overcome such perceived threat for dining out. The following hypotheses are proposed:

*H4a*: Perceived severity moderates the relationship between hedonic value and behavioral intention.

*H4b*: Perceived severity moderates the relationship between utilitarian value and behavioral intention.

*H4c*: Perceived severity moderates the relationship between social value and behavioral intention.

*H5a*: Perceived susceptibility moderates the relationship between hedonic value and behavioral intention.

*H5b*: Perceived susceptibility moderates the relationship between utilitarian value and behavioral intention.

*H5c*: Perceived susceptibility moderates the relationship between social value and behavioral intention.

## Methodology

### Research context: Dining in a restaurant in the new normal context of COVID-19

Due to their vulnerability to the pandemic crisis (Kim et al., 2021), restaurant and tourism industries are some of the most affected sectors, where governments, organizations, and academia have been actively seeking appropriate strategies to help the economic recovery (Liu et al., 2021a). Facing the perceived threat of COVID-19, consumers would perform more citizenship and participation behaviors to protect themselves, such as wearing a mask (Min et al., 2021). In Malaysia, diners might switch from dine-in to online food delivery if they perceive vulnerability due to COVID-19 situations (Mahmood et al., 2022). In India, the consumers in fine-dining restaurants look for not only food quality and price but also many hygienic practices at the restaurant (Lim et al., 2022). In Indonesia, food safety-related attitudes, subjective norms, and perceived behavioral control improve dining-out intention (Soon et al., 2021). In Korea, diners’ subjective norms and enjoyment encourage dining out, while psychological risks avoid it (Zhong et al., 2021). In China, the recovery process started from dining out to domestic travel, business or outbound tourism, and hospitality activities (iResearch, 2020). In that process, Chinese consumers learned new behavior patterns in response to the multiple waves of the pandemic (Puttaiah et al., 2020). Pan and Ha (2021), for example, reported that, during COVID-19, Chinese diners were satisfied with process service quality more than outcome service quality; this result was in contradiction to those obtained by traditional service studies prior to COVID-19. The changing patterns of consumers’ emotional experience and psychological states on their dining-out behaviors have not been well known in the literature (Hassan and Soliman, 2021; Kim et al., 2021), which could be crucial to the revival of the restaurant industry. In addition, coronavirus was not the last pandemic (BBC, 2020b), and the food and beverage industries would need to deal with other pandemics in the future (Zhong et al., 2021). Thus, it should be valuable to understand the emotions and perceptions that can drive consumers to dine out after a pandemic.

This paper defines a restaurant as a facility or dining room in a certain place that openly provides food and beverage services to the public, including local specialty restaurants, barbecue restaurants, seafood restaurants, hot pot restaurants, dining places in hotels, and banquet halls, but excluding fast-food restaurants, schools, and company canteens. In this way, restaurants that provide full services to consumers (Brown, 2020), who could consider all hedonic, utilitarian, and social values of dining in a restaurant (Ha and Jang, 2012), were selected. In addition, in this paper, dining out means that consumers dine in a restaurant rather than ordering delivery or takeaway.

### Measurements

All research constructs were measured using multiple items with a seven-point Likert scale, as shown in Table 1. The scales of positive and negative anticipated emotions were taken from Bagozzi and Dholakia (2002), Lee et al. (2012), and Tsai and Bagozzi (2014). The hedonic, utilitarian, and social value scales were adapted from Hyun et al. (2011), Sweeney and Soutar (2001), Babin et al. (1994), Park (2004), and Babin et al. (2005). The scales of perceived severity and susceptibility were adapted from Cahyanto et al. (2016) and Champion (1984). The behavioral intention scales were adapted from Perugini and Bagozzi (2001) and Lee et al. (2012). The measures were then verified through consumer interviews and a pretest conducted using the confirmatory factor analysis, as described below.

**Table 1:** The results of the measurement model.

Constructs	Items	Standardized factor loading	Cronbach’s α	AVE	CR
Positive anticipated emotion (PAE)	If I can dine in a restaurant, I will be:				
	1. Excited.	0.76***	0.895	0.638	0.898
	2. Glad.	0.858***			
	3. Satisfied.	0.715***			
	4. Happy.	0.839***			
	5. Delighted.	0.814***			
Negative anticipated emotion (NAE)	If I cannot dine in a restaurant, I will be:				
	1. Angry.	0.76***	0.909	0.630	0.911
	2. Disappointed.	0.738***			
	3. Worried.	0.863***			
	4. Sad.	0.865***			
	5. Frustrated.	0.761***			
	6. Depressed.	0.766***			
Hedonic value (HV)	1. Dining out was truly a joy.	0.806***	0.826	0.550	0.83
	2. Compared to other things, the experience of dining out was truly enjoyable.	0.72***			
	3. Dining out truly felt like an escape.	0.712***			
	4. I preferred going to the restaurant to feel good feeling.	0.725***			
Utilitarian value (UV)	1. Dining in a restaurant was pragmatic and economical.	0.713***	0.702	0.542	0.703
	2. The cost of eating in a restaurant was reasonable.	0.759***			
Social value (SV)	Dining out				
	1. Helped me get to know my companions.	0.676***	0.807	0.52	0.812
	2. Helped me get socially accepted in the group.	0.794***			
	3. Would make a good impression on other people.	0.700***			
	4. Helped to maintain the relationship with companions.	0.708***			
Perceived severity (PSEV)	1. The thought of COVID 19 scares me.	0.743***	0.862	0.682	0.865
	2. When I think about COVID 19 I feel nauseous.	0.91***			
	3. When I think about COVID 19 my heart beats faster.	0.817***			
Perceived susceptibility (PSUS)	1. My chances of getting COVID 19 are great.	0.801***	0.868	0.625	0.870
	2. My physical health makes it more likely that I will get COVID 19.	0.739***			
	3. I feel that my chances of getting COVID 19 in the future are good.	0.839***			
	4. There is a good possibility that I will get COVID 19.	0.781***			
Behavioral intention (BI)	1. I intend to dine out in the near future.	0.77***	0.882	0.603	0.883
	2. I am planning to dine out in the near future.	0.763***			
	3. I will invest time to dine out in the near future.	0.745***			
	4. I will invest money to dine out in the near future.	0.798***			
	5. I am willing to dine out in the near future.	0.805***			
χ^2^ = 1225.268, df = 499, *p* < 0.001, χ^2^/df = 2.455, RMR = 0.070, SRMR = 0.0412, RMSEA = 0.048, CFI = 0.938, IFI = 0.939, TLI = 0.931					

*** *p* < 0.001.

### Sampling and data collection

The questionnaire was developed in English and then translated into Chinese. To validate the instruments used in the current study, a backward-translation approach was adopted (Gallarza et al., 2015) and then two bilingual management professors were interviewed to examine the clarity and readability of the questionnaires. Finally, 10 Chinese restaurant consumers were interviewed and a pre-test was performed on 47 Chinese restaurant consumers (Ha and Jang, 2010) to complete the questionnaire design.

Given the impact of the COVID-19 pandemic, an online survey was conducted to avoid physical contact. A famous marketing research company in China, So jump, was employed for data collection (Jiang and Lau, 2021). Regarding the sample selection, considering the vital role of the hospitality industry in the economic development of big cities (China Statistical Yearbook, 2019), respondents from Beijing, Shanghai, Hangzhou, Shenzhen, and Guangzhou were selected as the study target. These cities were selected because their COVID-19 situations were largely improved in May 2020, with no new cases reported since April 1st, 2020, and the Chinese government had begun an orderly recovery effort in these cities (CSCI, 2020; Wen et al., 2020). In addition, only those respondents who had stable monthly income at the time of data collection were selected, as a stable household income could encourage full-service restaurant visits (French et al., 2010). Based on the two selection criteria, the research company randomly distributed online questionnaires to the potential respondents from May 31 to June 25, 2020, and a total of 800 questionnaires were collected. The respondents were assured regarding their anonymity and that no personal information would be disclosed. In addition, they were instructed that the study results were only for academic research purpose and that there were no right or wrong answers. Then, respondents who visited restaurants at least once after they were reopened were selected. The response quality was validated by having some items with reverse coding in the questionnaire. After removing invalid (e.g., respondents who gave inconsistent answers, selected the same rating for every item or adopted straight-lining, completed the questionnaires within a few minutes, and did not live in the targeted cities) and incomplete (e.g., the respondents who did not rate all items) questionnaires, 621 valid responses were used for subsequent data analysis.

### Sample profiles

The sample demographic information is summarized in Table 2, which is similar to that used in prior studies (Choi et al., 2020b). The majority of the respondents were married (71.5%) and lived with family members (81%). Approximately 46% of the respondents had an income of more than 10,000 RMB per month. Before the COVID-19 outbreak, all respondents dined in restaurants more than once a week, 49.3% dined out 2–3 times per week, and 34.5% dined out 4–5 times every week. After the outbreak and lockdowns, dining in a restaurant became much less frequent. Of them, 35.6 dined out equal to or less than once a week. The proportions of the respondents eating out 2–3 times (42.7%) or 4–5times (16.1%) a week were dramatically reduced. This showed that, even though the COVID-19 situation was largely recovered at the time of the study, it still discouraged dining-out behaviors. Thus, the survey data were appropriate for this study.

**Table 2:** Sample profile.

Variable	Level	Frequency	Valid %
Gender	Female	380	61.2
	Male	241	38.8
Marital status	Single	173	27.9
	Married	444	71.5
	Other (widowed, divorced etc.)	4	0.6
Residence status	Live alone	49	7.9
	Live with roommate	59	9.5
	Live with family members	503	81.0
	Others	10	1.6
Occupation	Professionals	248	39.9
	Business owner	23	3.7
	Service worker	23	3.7
	Office worker	241	38.8
	State workers	18	2.9
	Educators	37	6.0
	Freelancers	17	2.7
	Retiree	4	0.6
	Unemployed	1	0.2
	Other	9	1.4
Salary (RMB)	< 5,000	48	7.7
	5,000 ~ 8,000	139	22.4
	8,000 ~ 10,000	146	23.5
	10,000 ~ 15,000	181	29.1
	≥15,000	107	17.2
Education	<high school degree	24	3.9
	College graduate	56	9.0
	Undergraduate	455	73.3
	Postgraduate	86	13.8
Before the outbreak, frequency of dining out per week	≤1 time	0	0
	2–3 times	306	49.3
	4–5 times	214	34.5
	6–7 times	61	9.8
	8–10 times	20	3.2
	>10 times	20	3.2
Frequency of dining out after restaurants reopened	≤1 time	221	35.6
	2–3 times	265	42.7
	4–5 times	100	16.1
	6–7 times	23	3.7
	8–10 times	7	1.1
	>10 times	5	0.8

## Results

### Measurement model

Structural equation modeling (SEM) using AMOS 24 was adopted to analyze the proposed hypotheses. A confirmatory factor analysis (CFA) was performed. As shown in Table 1, the results of CFA presented a good model fit (value of *p* < 0.001, χ^2^/df = 2.455, SRMR = 0.0412, RMSEA = 0.048, CFI = 0.938, IFI = 0.939, TLI = 0.931). All Cronbach’s alpha and composite reliability (CR) values of the constructs were higher than their threshold of 0.7. The average variance extracted (AVE) values ranged from 0.520 to 0.682. All squared roots of the AVE values were larger than the corresponding correlation coefficients (Fornell and Larcker, 1981), as shown in Table 3.

**Table 3:** Correlations and square roots of average variance extracted (AVE).

	Mean	SD.	PAE	NAE	HV	UV	SV	PSEV	PSUS	BI
Positive anticipated emotion (PAE)	5.235	0.972	**0.799**							
Negative anticipated emotion (NAE)	3.369	1.237	0.395**	**0.794**						
Hedonic value (HV)	5.350	0.943	0.706**	0.391**	**0.742**					
Utilitarian value (UV)	5.109	1.073	0.376**	0.240**	0.446**	**0.736**				
Social value (SV)	5.365	0.942	0.355**	0.213**	0.441**	0.333**	**0.721**			
Perceived severity (PSEV)	4.303	1.394	−0.013	0.097*	0.030	−0.006	0.061	**0.826**		
Perceived susceptibility (PSUS)	2.192	1.049	−0.087*	0.040	−0.110**	−0.090*	−0.033	0.299**	**0.791**	
Behavioral intention (BI)	5.669	0.911	0.630**	0.271**	0.601**	0.429**	0.306**	−0.050	−0.122**	**0.777**

Square root of AVE is on the diagonal and shown in bold. Correlations of paired constructs are under the AVE. Correlation is significant at the 0.01 level (**) and 0.05 level (*) (two-tailed).

### Non-response bias and common method bias testing

To examine the non-response bias, early and late respondent tests were conducted, and no statistically significant results were obtained. To assess CMB, Harman’s single-factor test was performed, and the first single factor accounted for 28.8% of the total variance. In addition, the test results of an unmeasured latent method factor analysis (*p* < 0.001, χ^2^/df = 1.948, SRMR = 0.056, RMSEA = 0.039, CFI = 0.964, IFI = 0.964, TLI = 0.956) were similar to those of the original CFA, suggesting that CMB was not severe in this study (Podsakoff et al., 2003).

### Structural model

**Figure 2:**
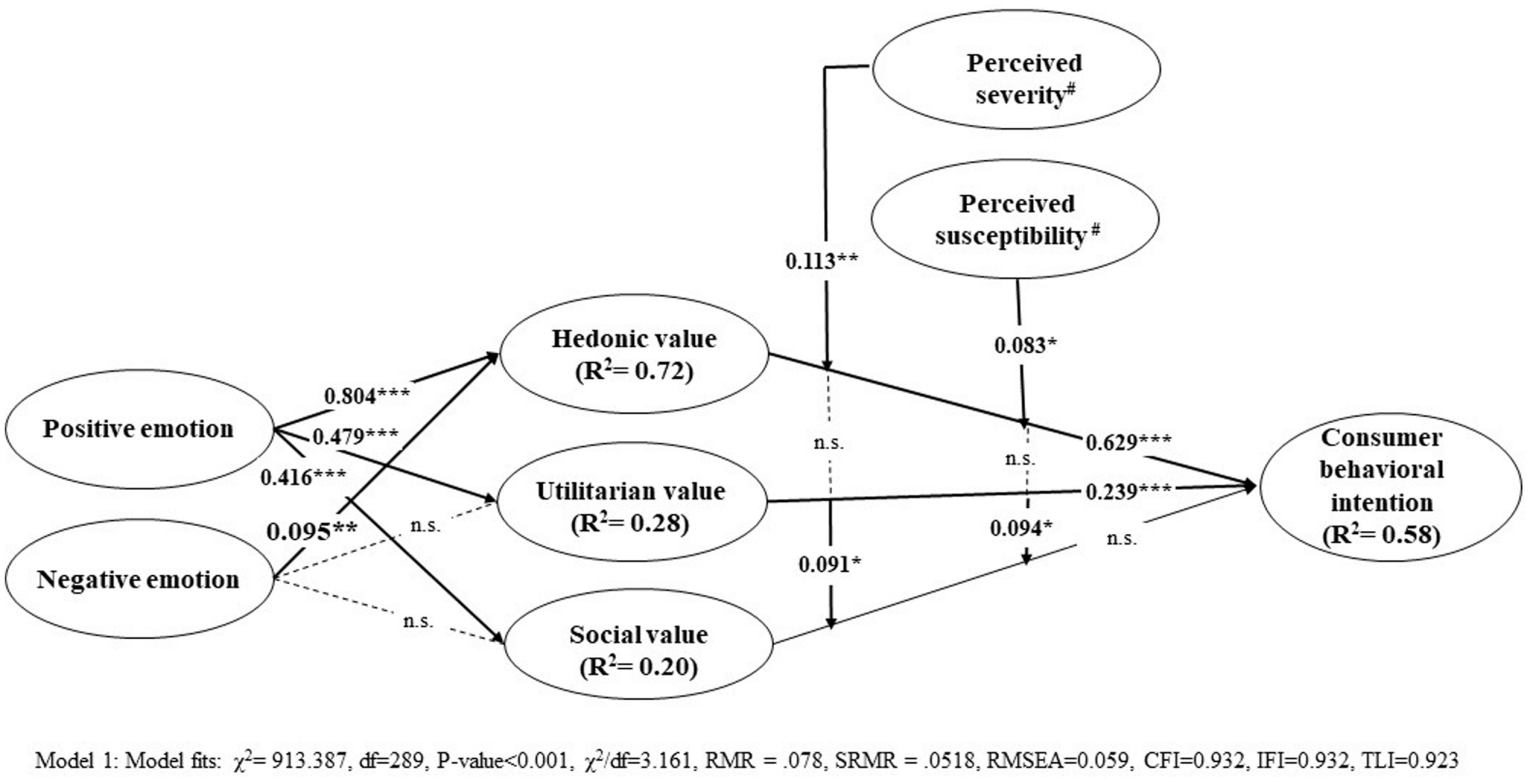
Result of research model. ***p-value <0.001; **p-value <0.01; *p-value <0.05. n.s. not statistically significant. Significant correlation values at the value of *p* < 0.05 were highlighted in bold. ^#^ Model fits for Model 2 and 3 of perceived severity and perceived susceptibility respectively are reported in Table 5.

The research model with all proposed hypotheses was tested by maximum likelihood estimation in SEM, as shown in Figure 2. The model had a satisfactory fit (χ^2^ = 913.387, df = 289, *p* < 0.001, χ^2^/df = 3.161, RMR = 0.078, SRMR = 0.0518, RMSEA = 0.059, CFI = 0.932, IFI = 0.932, TLI = 0.923). The magnitude of income change during the COVID-19 pandemic, gender, and education level were added as three control variables, and the results did not show any significant changes in the overall model (χ^2^ = 1040.911, df = 358, *p* < 0.001, χ^2^/df = 2.908, RMR = 0.072, SRMR = 0.0527, RMSEA = 0.055, CFI = 0.926, IFI = 0.927, TLI = 0.916). The structural model results are reported in Table 4. The results showed that the relationships between the positive anticipated emotions and perceived values [i.e., hedonic value (*β* = 0.804, *t* = 16.452), utilitarian value (*β* = 0.479, *t* = 8.079), and social value (*β* = 0.416, *t* = 7.707)] were all statistically significant, supporting H1a–c. The effect of negative anticipated emotions on hedonic value (*β* = 0.095, *t* = 2.721) was statistically significant, but not on utilitarian value (*β* = 0.095, *t* = 1.852) and social value (*β* = 0.065, *t* = 1.346). The results supported H2a but not H2b–c. Hedonic value (*β* = 0.629, *t* = 12.016) and utilitarian value (*β* = 0.239, *t* = 4.977) were positively correlated with behavioral intention but not social value (*β* = −0.017, *t* = −0.42). Thus, H3a–b were supported, but not H3c was supported.

**Table 4:** Statistical results of direct, indirect, and mediation effects.

Relationships	Model 1		
	Direct effects	*t* value	Indirect effects
H1a Positive anticipated emotion ➔ hedonic value.	**0.804** ***	**16.452**	
H1b Positive anticipated emotion ➔ utilitarian value.	**0.479** ***	**8.079**	
H1c Positive anticipated emotion ➔ social value.	**0.416** ***	**7.707**	
H2a Negative anticipated emotion ➔ hedonic value.	**0.095** **	**2.721**	
H2b Negative anticipated emotion ➔ utilitarian value.	0.095	1.852	
H2c Negative anticipated emotion ➔ social value.	0.065	1.346	
H3a Hedonic value ➔ behavioral intention.	**0.629** ***	**12.016**	
H3b Utilitarian value ➔ behavioral intention.	**0.239** ***	**4.977**	
H3c Social value ➔ behavioral intention.	−0.017	−0.428	
**Statistical results of mediation effects**			
Positive anticipated emotion ➔ behavioral intention			**0.613** **
Negative anticipated emotion ➔ behavioral intention			**0.081** **
Positive anticipated emotion ➔ hedonic value ➔ behavioral intention			**0.462** ***
Positive anticipated emotion ➔ utilitarian value ➔ behavioral intention			**0.104** **
Positive anticipated emotion ➔ social value ➔ behavioral intention			−0.006
Negative anticipated emotion ➔ hedonic value ➔ behavioral intention			**0.046** **
Negative anticipated emotion ➔ utilitarian value ➔ behavioral intention			**0.018** *
Negative anticipated emotion ➔ social value ➔ behavioral intention			−0.001
Model fits: χ^2^ = 913.387, df = 289, *p* < 0.001, χ^2^/df = 3.161, RMR = 0.078, SRMR = 0.0518, RMSEA = 0.059, CFI = 0.932, IFI = 0.932, TLI = 0.923			

Significant correlation values at the value of *p* < 0.05 were highlighted in bold.

*** *p* < 0.001,

** *p* < 0.01,

* *p* < 0.05.

In SEM, a multi-group analysis was used to examine the nonlinear effect if the moderator was categorical. However, as the moderators of this study were two continuous variables, a multi-group analysis might not be appropriate due to the loss of statistical power (Lam et al., 2004). Thus, an interaction effect approach was used (Rasoolimanesh et al., 2021). As such, Ping Jr’s (1995) single indicant method was adopted, which created single indicants for the interaction constructs of perceived severity and susceptibility on the relationship of the three perceived values on behavioral intention. This approach could avoid redundant variables with complex parameter constraints and has been used in many recent hospitality studies (e.g., Han and Hwang, 2019; Lei et al., 2020; Yoo and Cho, 2021; Horng et al., 2022). Following Ping Jr’s (1995) single indicant approach, all indicators of the latent variables were mean-centered, a single indicator for the latent product method was calculated, and the structural equation model with the two focal variables and their interaction term was specified. The procedure followed De Luca and Atuahene-Gima’s (2007) study with the following equations:


$$\lambda_{xz}=\left(\lambda_{x1}+\ldots +\lambda_{xn}\right)\left(\lambda_{z1}+\ldots +\lambda_{zm}\right),$$

$$\begin{array}{c} \theta_{\varepsilon xz}={\left(\lambda_{x1}+\ldots +\lambda_{xn}\right)}^{2}Var\left(X\right)\left(\theta_{\varepsilon z1}+\cdots +\theta_{\varepsilon zm}\right)\\ \;+{\left(\lambda_{z1}+\ldots +\lambda_{zm}\right)}^{2}Var\left(Z\right)\left(\theta_{\varepsilon z1}+\cdots +\theta_{\varepsilon xn}\right)\\ \;+\left(\theta_{\varepsilon z1}+\cdots +\theta_{\varepsilon zm}\right)\left(\theta_{\varepsilon z1}+\cdots +\theta_{\varepsilon xn}\right) \end{array}$$

Note: X and Z represent each pair of latent variables, and X_1_-Xn and Z_1_-Zm represent their indicators.

The results presented in Table 5 show that the interactive effects of perceived severity and susceptibility on the relationships between hedonic and social values on behavioral intention were statistically significant, but not utilitarian value. The results supported H4a, H4c and H5a, H5c, but not H4b and H5b.

**Table 5:** Statistical results of moderation effects.

Relationships		Model 2		Model 3	
		β	*t* value	β	*t* value
Perceived severity ➔ behavioral intention		**−0.100** **	**−3.016**		
H4a: Perceived severity* **hedonic value** ➔ **behavioral intention**		**0.113** **	**3.027**		
H4b: Perceived severity*utilitarian value ➔ behavioral intention		−0.022	−0.556		
H4c: Perceived severity*social value ➔ **behavioral intention**		**0.091** *	**2.513**		
Perceived susceptibility ➔ behavioral intention				−0.064	−1.874
H5a: Perceived susceptibility*hedonic value ➔ **behavioral intention**				**0.083** *	**2.266**
H5b: Perceived susceptibility*utilitarian value ➔ behavioral intention				0.011	0.266
**H5c: Perceived susceptibility** *social value ➔ **behavioral intention**				**0.094** *	**2.450**
Model fits		χ^2^ = 1545.81, df = 451, *p* < 0.001, χ^2^/df = 3.428, SRMR = 0.0623, RMR = 6.379, RMSEA = 0.063, CFI = 0.896, IFI = 0.897, TLI = 0.886		χ^2^ = 1401.362, df = 481, *p* < 0.001, χ^2^/df = 2.913, SRMR = 0.0564, RMR = 3.804, RMSEA = 0.056, CFI = 0.915, IFI = 0.915, TLI = 0.907	

** *p* < 0.01,

* *p* < 0.05. Significant correlation values at the value of *p* < 0.05 were highlighted in bold.

Danielsoper’s (2016) post-hoc statistical power calculator was used to test the power of the study model (Khan et al., 2017). The power analysis was performed considering 1 – β as a function of significant level α, sample size, and observed R^2^. The results showed that the observed statistical power of behavioral intention and hedonic, utilitarian, and social values was equal to one, showing that the model holds adequate power (>0.85; Cohen, 1992).

## Discussion

Consistent with the hypotheses, this study finds that positive anticipated emotions are significantly associated with all three types of perceived value, with the greatest effect on hedonic value in the pandemic context. This result agrees with those obtained by previous studies in which people in a positive emotional state were found to be motivated to maintain their good mood and thus evaluate the dining experience more positively (e.g., Wegener et al., 1995; Park, 2004; Babin et al., 2005; Song and Qu, 2017). In the context of this study, this shows that when consumers have a positive emotional anticipation to dine in a restaurant after the social distancing measures are relaxed, they would experience higher perceived value from the dining-out experience, in terms of entertaining and a pleasant dining atmosphere, low price, service quality, menu variety, benefits, and social interactions with other people (e.g., Song and Qu, 2017). This may help explain why revenge spending behaviors (Derousseau, 2021) could be affected by the positively biased perceived value of dining out. This needs to be studied further.

Partially consistent with the research hypotheses, negative anticipated emotions are found to have a significant influence on hedonic value only. While individuals tend to avoid pain or negative emotions (Higgins, 1997), negative emotions may have a much smaller effect on product evaluation (Bagozzi et al., 1999). The insignificant effect of negative anticipated emotions on utilitarian and social values correspond to the findings of Babin et al. (2005) and Lo and Wu (2014). It is possible that dining in a restaurant is a kind of concrete intention or action, and the connection between dining in a restaurant and other higher-level ends (e.g., feel well or enjoy time) is readily inferred (Perugini and Bagozzi, 2001). Dining out can be seen as an achievable behavior during the early recovery stage of COVID and is, in itself, associated with positive emotions (Foroudi et al., 2021). The negative emotional consequences of not dining out may not be sufficiently significant to alter the consumer’s evaluation of certain perceived values (i.e., utilitarian and social values) to engage in dining-out behavior in this context (Perugini and Bagozzi, 2001; Foroudi et al., 2021). Alternatively, when consumers feel negative anticipated emotions, they may hold doubts and suspend the product evaluation (Wood and Moreau, 2006). Accordingly, customers with negative anticipated emotions were suspected to postpone the evaluation of dining experiences from social and utilitarian perspectives. Thus, negative anticipated emotions haves no or a weaker effect on these two perceived values in this study.

Furthermore, hedonic and utilitarian values are found to be positively correlated with consumers’ behavioral intention to dining in a restaurant (Ha and Jang, 2010; Hyun et al., 2011; Song and Qu, 2017), and hedonic value plays a greater role. As the targeted restaurants mainly include local specialty restaurants, barbecue restaurants, seafood restaurants, and hot pot restaurants but exclude fast-food restaurants, the customers may look for hedonic value, such as pleasant dining atmosphere, gorgeous food décor, and service quality more than the other perceived values (Lee and Hwang, 2011; Brown, 2020). Thus, hedonic value is more important than utilitarian value in this study (Park, 2004).

Inconsistent with the expectation of this study, social value has no direct effect on dining out intention, indicating that social value was not a crucial factor in influencing consumers’ dining-in intention at the time of the study. It is possible that, in the early recovery of COVID-19, customers consciously maintained social distancing, reducing physical contact and social interactions with other people (Wen et al., 2020; Itani and Hollebeek, 2021). Thus, even if they dine out, they may eat alone or maintain minimal physical contact with other people or non-family members (Wen et al., 2020; Foroudi et al., 2021). In addition, as the study’s data show that most of the respondents live with their family members or roommates, the social value of dining out may not be critical for them (Table 2). Thus, the direct effect of social value on behavioral intention is insignificant in this study.

Finally, the study explored that when consumers perceive a high level of perceived threat from the COVID-19 pandemic, consumers’ hedonic and social values of dining in restaurants have a stronger positive effect on their behavioral intention. This result shows that, during the early recovery period of COVID-19, the greater the psychological pressure consumers bear, the more perceived value they need to overcome the threats to dine out. When consumers perceive a high level of threat, they may not be very concerned about the price of dining out. Instead, they may highly value a pleasant dining atmosphere and social relationships. Combining this finding with the result of hypothesis H3c suggests that social value, in general, does not affect behavioral intention. But, it becomes significant when the consumers are stressful about the perceived threat from COVID-19 that treasures social support (e.g., meeting people in restaurants) to maintain their physical and psychological health (Klümper and Sürth, 2021).

## Conclusion

This study explored the impact of anticipated emotions on perceived values and future revisiting intentions in the restaurant industry in the context of early COVID-19 recovery. As shown in Table 6, the results suggest that positive anticipated emotions (e.g., positive affect and joy) can influence hedonic, utilitarian, and social perceptions of the dining experiences in restaurants, which, in turn, affect consumers’ dining-out intention. Negative anticipated emotions (e.g., anger, sadness, and fear) can affect hedonic value but not utilitarian and social values. The relationships between hedonic and social values on future visiting intentions are strengthened if consumers perceive a higher level of COVID-19 threat. The findings of the present study distill theoretical and practical implications, as discussed below.

**Table 6:** Results of hypothesis testing.

Hypothesis	Support
H1a Positive anticipated emotion is positively associated with hedonic value.	Yes
H1b Positive anticipated emotion is positively associated with utilitarian value.	Yes
H1c Positive anticipated emotion is positively associated with social value.	Yes
H2a Negative anticipated emotion is positively associated with hedonic value.	Yes
H2b Negative anticipated emotion is positively associated with utilitarian value.	No
H2c Negative anticipated emotion is positively associated with social value.	No
H3a Hedonic value is positively associated with behavioral intention.	Yes
H3b Utilitarian value is positively associated with behavioral intention.	Yes
H3c Social value is positively associated with behavioral intention.	No
H4a Perceived severity moderates the relationship between hedonic value and behavioral intention.	Yes
H4b Perceived severity moderates the relationship between utilitarian value and behavioral intention.	No
H4c Perceived severity moderates the relationship between social value and behavioral intention.	Yes
H5a Perceived susceptibility moderates the relationship between hedonic value and behavioral intention.	Yes
H5b Perceived susceptibility moderates the relationship between utilitarian value and behavioral intention.	No
H5c Perceived susceptibility moderates the relationship between social value and behavioral intention.	Yes

### Theoretical contributions

The current study has several theoretical contributions. First, this study contributes to the emotion literature by verifying the role of anticipated emotions affected by a social stimulus or a forced behavior (i.e., the outbreak of COVID-19) in understanding consumer dining-out behavior (Jeong et al., 2021; Liu et al., 2021a). The results of this study show that anticipated emotions are an important personal input that affects the perceived value of customers, leading to their behavioral intention of dining out under this new normal context. It provides a more comprehensive view of how a socially stimulated emotion affects consumer behavioral intention (Hassan and Soliman, 2021; Zhong et al., 2021). In particular, consumers with positive anticipated emotions to dine out perceive higher hedonic, utilitarian, and social values to dine in a restaurant. Consumers with negative anticipated emotions to not dine out perceive higher hedonic value to dine in. These findings are novel in the literature.

Moreover, this paper provides new evidence to support the anticipated emotion literature that positive and negative anticipated emotions are related but distinct constructs, which should be examined individually (Bagozzi et al., 1998; Liu and Jang, 2009; Foroudi et al., 2021). It shows the distinct effects of positive and negative anticipated emotions on perceived value. Consistent with existing literature (e.g., Babin et al., 2005; Wood and Moreau, 2006; Lo and Wu, 2014; Ahn and Kwon, 2020), the effect of the anticipated emotions on dining behaviors appear to be stronger for positive anticipated emotions than the negative emotions, and positive emotions may be a more stable factor affecting the evaluation of a service.

Extending to the TPB/MGB concepts, this study shows that emotions play a critical role in consumption (Bagozzi et al., 1998, 2016; Richetin et al., 2008; Xie et al., 2013; Parkinson et al., 2018). While the roles of TPB variables on behavioral intention are increasingly examined in tourism and hospitality literature with a focus on sustainability, the internet, and social media (Ulker-Demirel and Ciftci, 2020), the affective elements of behavioral intention, proposed by MGB, are not well-tested in the literature on COVID-19 situations (Chiu and Cho, 2022). This study can address this by showing that positive and negative anticipated emotions can distinctively affect behavioral intention, mediated by perceived value and moderated by perceived threats. This finding supports existing studies that anticipated emotions motivate volitional processes that perform directive goals or intentions (Bagozzi et al., 1998, 2016). While this study does not control for the TPB variables, the anticipated emotions are suspected to be different from affective attitudes (Conner et al., 2013), which need to be separately examined in behavioral intention (Parkinson et al., 2018). Anticipated emotions can affect how consumers evaluate or perceive product offerings, resulting in consumption intention (Hyun et al., 2011; Lo and Wu, 2014). Future research may integrate the TPB/MGB model with perceived value to verify the findings of this study.

Second, this study contributes to the hospitality literature by exploring the roles of perceived value in consumer dining-in behavior in the context of COVID-19 recovery (Pai et al., 2021; Touni et al., 2022). This study extends previous research by simultaneously assessing the hedonic, utilitarian, and social values of dining in a restaurant (Ha and Jang, 2010; Yu et al., 2013; Song and Qu, 2017). This paper also provides new evidence on how consumer emotions and perception can encourage dining-out behaviors during the early recovery of COVID-19. In this way, this paper answers the calls for extensive consumer studies on how psychological factors impact consumer behaviors due to COVID-19 (Kirk and Rifkin, 2020; Blazquez-Resino et al., 2022). Additionally, as the mediating roles of the three perceived values are examined together in a single test, hedonic value functions are shown to be the greatest contributor to dining-out intention, followed by utilitarian value. Social value has no significant effect in this context. This result shows that while consumers remain wary of the pandemic, hedonic and utilitarian values play vital roles in driving customers to dine in a restaurant (Wen et al., 2020; Zhong et al., 2021), but not social value, which involves socialization in a dining room. In this way, this research enriches the existing literature on perceived value in the context of COVID-19.

Finally, the moderating effect of perceived severity and susceptibility on the relationship between perceived value and behavioral intention is verified. Existing literature usually examines perceived threat as an antecedent of customer behaviors (e.g., stay-at-home or hoarding; Laato et al., 2020; Kim et al., 2021). This study, in contrast, propose that such threat could be a moderator of behavioral intention (Campbell and Goodstein, 2001; Kim, 2020). It is found that when consumers have a higher level of perceived threat of the pandemic, utilitarian values (e.g., product price) are not a concern. Instead, hedonic (e.g., enjoying the dining place) and social (e.g., seeing people) values play a more critical role in motivating the intention to dine out. It can be explained that consumers have a strong need to emotionally and socially relieve their heightened pressure and loneliness and transform their mood from the COVID-19 pandemic. This new empirical finding is not available from prior literature and requires further research.

### Managerial implications

The current research provides three new insights into the business recovery of the restaurant industry from a consumer psychology perspective. First, both positive (e.g., like to dine out) and negative (e.g., disappointed to not be able to dine out) anticipated emotions positively affect consumers’ perceived value of dining out. This indicates that consumers’ anticipated emotions help improve the perceived value of dining in a restaurant. Furthermore, the results show that positive anticipated emotions affect all three perceived values with a greater impact than negative anticipated emotions. Therefore, during the early stage of recovery, restaurant managers are recommended to adopt various marketing techniques to promote the positive emotions of dining in a restaurant. For example, following the concept of goal-directed emotions that the anticipated emotion can be affected by the subjective appraisal of all characteristics of the environment (Bagozzi et al., 1998), restaurants may stimulate consumer consumption emotions and desire for dining out by creating a favorable dining atmosphere through social media and online peer reviews (Hlee et al., 2019; Haas et al., 2022). The restaurants should build up the consumer’s certainty by showing them that the dining place is clean and safe. Cashless payment methods can be used to ensure safety. The menu should be simplified, and food should be easy to order online. In addition, they could advertise the missed opportunities of not to dine out. When the anticipated emotions are formed, consumers may perceive a higher value of dining out and thus show more intention to dine out.

Second, hedonic and utilitarian values significantly affect consumers’ dining-out intention in the new normal context, with hedonic value having the greatest effect. Thus, for business recovery, restaurant managers should prioritize the emotional experience of dining in a restaurant (e.g., décor, physical environment, service, and food quality; Hyun et al., 2011; Gallarza et al., 2015; Chen and Peng, 2018). Meanwhile, the role of utilitarian value of dining out should not be ignored. Restaurants should appropriately emphasize the usefulness of consumption in efficient, task-related, functional, and economic aspects (e.g., discounts, good price, worthwhile, and a variety of dishes; Kim et al., 2013a,b) when consumers dine in their restaurants. Instead, the restaurants may not promote the social elements of dining in a restaurant and keep the social distancing practices in place (Fantozzi, 2020; Chuah et al., 2022).

Finally, consumers’ perceived threat of the pandemic affects their intention to dining in a restaurant by adjusting the impact of perceived value on dining-out intention. Restaurant operators or service providers are thus advised to keep monitoring the pandemic situation; provide appropriate remedies, such as hygiene and safety measures (Kim et al., 2021); and understand consumers’ cognition of the outbreak. They may provide timely release of positive information related to the pandemic (e.g., the decline in the number of confirmed cases, regional risk level, or active measures of the government; Zhong et al., 2021) and restaurant countermeasures (e.g., hygiene and disinfection conditions; Liu et al., 2021a) that show dining out as a safe and achievable consumption activity to reduce the consumer’s perceived threat of dining out. Restaurant managers can also reduce the perceived threat by providing sanitizers, setting safe layouts and sufficient ventilation, using new ordering technologies, regulating their employees to manage their personal hygiene, and practicing an appropriate level of social distancing (Fantozzi, 2020; Chuah et al., 2022; Sardar et al., 2022). As social media plays a significant role in influencing consumers’ level of perceived threat (McCarthy et al., 2006; Sánchez-Cañizares et al., 2021), governments and restaurant managers should convey positive and favorable information about dining out and managing potential diners’ perception of threat. According to the level of perceived threat, managers could also appropriately prioritize their resources to work on different perceived values in order to encourage dining-in behaviors. As coronavirus is not the last pandemic (BBC, 2020a,b), the implications of this study could remain useful even after the pandemic (Zhong et al., 2021). Thus, managers should be concerned about how consumer emotions and perception affect diners after a pandemic so that they can effectively meet consumers’ needs in similar situations in the future.

### Limitations and future research

This study has several limitations for further studies. First, the current study was limited to the selected research constructs. Although anticipated emotions are a significant factor that impacts behavior intention, other factors from TPB/GMB are crucial as well. Even if they are already reported in the literature (Foroudi et al., 2021), future research may include them in model development to further verify the findings of this study. As the first empirical study to verify the effect of anticipated emotions on the three perceived values, this study was limited to the empirical setting. Replicate studies should be conducted to verify the study results. In addition, as this study examined the broad view of anticipated emotions (i.e., positive and negative anticipated emotions) on behavioral intention, it could not be specified how different types of discrete emotions, such as regret, desire, or fear, affect the behavior changes (Roseman, 1991; Kim et al., 2021). Further studies may try to examine them in response to the pandemic. Second, we collected data after the early recovery of COVID-19. Thus, we could not verify the changes in perceived value or emotions before and after COVID-19. Future research may try to address this issue. Third, this study did not control for the restaurant types, which could affect how the research constructed should be evaluated. However, as stated, the study roughly selected to study full-service restaurants (Brown, 2020). Future research may replicate this study by comparing the results of luxury restaurants against casual restaurants (Hlee et al., 2019). Fourth, this study was limited to measurement selection and design. There were many human emotion measures (e.g., Geneva emotion wheel or multidimensional emotion questionnaire). While the study adopted a common measure in anticipated emotion studies, the findings of this study were limited to the measures used. Similarly, while the survey instrument referred the respondents to answer the questions according to the general perception of dining experience after COVID-19, it did not specify a single restaurant experience. Future research may use different types of emotion measures or referrals to verify these findings. Fifth, similar to other online survey studies, the findings of this study might be limited to the respondents’ sub-optimal responses. Finally, the survey subjects of this study were restaurant consumers in several big cities in China, which could not represent the social distancing situations of other countries. Future research can verify the theoretical framework in different countries and regions, thus generalizing the results of this study.

## Data availability statement

The raw data supporting the conclusions of this article will be made available by the authors, without undue reservation.

## Ethics statement

Ethical review and approval was not required for the study on human participants in accordance with the local legislation and institutional requirements. Written informed consent for participation was not required for this study in accordance with the national legislation and the institutional requirements.

## Author contributions

YJ contributed to the conceptualization, methodology, investigation, and formal analysis. AL contributed to the conceptualization, data collection, and funding acquisition. All authors contributed to the article and approved the submitted version.

## Funding

This work was supported by the National Research Foundation of Korea Grant funded by Korean Government (MOE; NRF-2016S1A2A2912137). This work was partially supported by the Key Laboratory of Multidisciplinary Management and Control of Complex Systems of Anhui Higher Education Institutes, Anhui University of Technology (No. RZ2200000691), and by Humanities and Social Sciences Research Project of Higher Education Institutions in Anhui Province (Key Project) (No.2022AH050268).

## Conflict of interest

The authors declare that the research was conducted in the absence of any commercial or financial relationships that could be construed as a potential conflict of interest.

## Publisher’s note

All claims expressed in this article are solely those of the authors and do not necessarily represent those of their affiliated organizations, or those of the publisher, the editors and the reviewers. Any product that may be evaluated in this article, or claim that may be made by its manufacturer, is not guaranteed or endorsed by the publisher.

## Footnotes

^1^The study conducted an additional literature search to verify the statement of this study by using the following query to search articles in Web of Science on October 23, 2022. ALL = (COVID-19 OR coronavirus OR COVID) AND ALL = ("perceived value*") AND ALL = ("behavioral intention*" OR "behavioural intention*") AND ALL = (threat*). Among all SSCI/SCI journals, only three articles were found. Two were related to cruise transportation and one was related to hotels. While this search might be limited by the selection of search keywords or the WoS database, this result suggests that there is insufficient research in this area.
